# Associations between Arsenic Species in Exfoliated Urothelial Cells and Prevalence of Diabetes among Residents of Chihuahua, Mexico

**DOI:** 10.1289/ehp.1307756

**Published:** 2014-06-27

**Authors:** Jenna M. Currier, María C. Ishida, Carmen González-Horta, Blanca Sánchez-Ramírez, Lourdes Ballinas-Casarrubias, Daniela S. Gutiérrez-Torres, Roberto Hernández Cerón, Damián Viniegra Morales, Francisco A. Baeza Terrazas, Luz M. Del Razo, Gonzalo G. García-Vargas, R. Jesse Saunders, Zuzana Drobná, Rebecca C. Fry, Tomáš Matoušek, John B. Buse, Michelle A. Mendez, Dana Loomis, Miroslav Stýblo

**Affiliations:** 1 Curriculum in Toxicology, University of North Carolina at Chapel Hill, Chapel Hill, NC, USA; 2 Programa de Maestría en Ciencias en Biotecnología, Facultad de Ciencias Químicas, Universidad Autónoma de Chihuahua, Chihuahua, México; 3 Colegio de Médicos Cirujanos y Homeópatas del Estado de Chihuahua, A.C., Mexico; 4 Departamento de Toxicología, Centro de Investigación y de Estudios Avanzados del Instituto Politécnico Nacional, México Distrito Federal, México; 5 Facultad de Medicina, Universidad Juárez del Estado de Durango, Gómez Palacio, Durango, México; 6 Department of Nutrition, and; 7 Department of Environmental Sciences and Engineering, UNC Gillings School of Global Public Health, Chapel Hill, North Carolina, USA; 8 Institute of Analytical Chemistry of the Academy of Sciences of the Czech Republic, v. v. i., Brno, Czech Republic; 9 UNC School of Medicine, Chapel Hill, North Carolina, USA; 10 Carolina Population Center, and; 11 Lineberger Cancer Center, University of North Carolina at Chapel Hill, Chapel Hill, North Carolina, USA; 12 Monographs Section, International Agency for Research on Cancer, Lyon Cedex, France

## Abstract

Background: A growing number of studies link chronic exposure to inorganic arsenic (iAs) with the risk of diabetes. Many of these studies assessed iAs exposure by measuring arsenic (As) species in urine. However, this approach has been criticized because of uncertainties associated with renal function and urine dilution in diabetic individuals.

Objectives: Our goal was to examine associations between the prevalence of diabetes and concentrations of As species in exfoliated urothelial cells (EUC) as an alternative to the measures of As in urine.

Methods: We measured concentrations of trivalent and pentavalent iAs methyl-As (MAs) and dimethyl-As (DMAs) species in EUC from 374 residents of Chihuahua, Mexico, who were exposed to iAs in drinking water. We used fasting plasma glucose, glucose tolerance tests, and self-reported diabetes diagnoses or medication to identify diabetic participants. Associations between As species in EUC and diabetes were estimated using logistic and linear regression, adjusting for age, sex, and body mass index.

Results: Interquartile-range increases in trivalent, but not pentavalent, As species in EUC were positively and significantly associated with diabetes, with ORs of 1.57 (95% CI: 1.19, 2.07) for iAs^III^, 1.63 (1.24, 2.15) for MAs^III^, and 1.31 (0.96, 1.84) for DMAs^III^. DMAs/MAs and DMAs/iAs ratios were negatively associated with diabetes (OR = 0.62; 95% CI: 0.47, 0.83 and OR = 0.72; 95% CI: 0.55, 0.96, respectively).

Conclusions: Our data suggest that uncertainties associated with measures of As species in urine may be avoided by using As species in EUC as markers of iAs exposure and metabolism. Our results provide additional support to previous findings suggesting that trivalent As species may be responsible for associations between diabetes and chronic iAs exposure.

Citation: Currier JM, Ishida MC, González-Horta C, Sánchez-Ramírez B, Ballinas-Casarrubias L, Gutiérrez-Torres DS, Hernández Cerón R, Viniegra Morales D, Baeza Terrazas FA, Del Razo LM, García-Vargas GG, Saunders RJ, Drobná Z, Fry RC, Matoušek T, Buse JB, Mendez MA, Loomis D, Stýblo M. 2014. Associations between arsenic species in exfoliated urothelial cells and prevalence of diabetes among residents of Chihuahua, Mexico. Environ Health Perspect 122:1088–1094; http://dx.doi.org/10.1289/ehp.1307756

## Introduction

Arsenic (As), and specifically its inorganic forms (iAs) [arsenite (iAs^III^) and arsenate (iAs^V^)], are naturally occurring drinking-water contaminants. Epidemiologic evidence (James et al. 2013; Kuo et al. 2013; Maull et al. 2012; Pan et al. 2013; Wang et al. 2014), including a prospective study (Kim et al. 2013), has linked chronic iAs exposure with the risk of diabetes mellitus. Several mechanisms by which iAs exposure can disrupt glucose homeostasis have been proposed (Maull et al. 2012). Trivalent iAs^III^ and the trivalent methylated arsenicals that are produced in the course of iAs metabolism—methylarsonite (MAs^III^) and dimethylarsinite (DMAs^III^)—play key roles in these mechanisms (Douillet et al. 2013; Fu et al. 2010; Paul et al. 2007, 2008). However, assessing the association between these arsenicals and risk of diabetes in population studies has been a major challenge. This is because iAs^III^—and particularly MAs^III^ and DMAs^III^—are unstable in human urine, which has been traditionally used in assessing iAs exposure and metabolism (Del Razo et al. 2011; Gong et al. 2001). An additional challenge is associated with quantification of iAs metabolites in urine, specifically with using urinary creatinine as a factor to adjust for variation in urine dilution. Urinary creatinine concentration is influenced by various factors, including age, sex, health status, ethnicity, body mass index (BMI), fat-free mass, and time of collection (Barr et al. 2005; Boeniger et al. 1993; Mahalingaiah et al. 2008). In addition, adjusting for creatinine may be inappropriate for individuals with compromised renal function, including people with diabetes (Jerums et al. 2010). More importantly, iAs exposure may lead to an increased excretion of creatinine (Nermell et al. 2008). Thus, the analysis of iAs metabolites in body fluids other than urine, such as in cells or tissues, may provide a better measure of iAs exposure.

We have previously demonstrated the feasibility of As analysis in human cells, including exfoliated urothelial cells (EUC). In 2008, we used hydride generation (HG)–cryotrapping (CT)–atomic absorption spectrometry (AAS) to measure concentrations of total iAs (iAs^III + V^), MAs (MAs^III + V^), and DMAs (DMAs^III + V^) in EUC isolated from the urine of residents of the Zimapan region in Mexico (Hernández-Zavala et al. 2008b). Because of small sample sizes, we could not perform the oxidation state-specific analysis to distinguish between As^III^ and As^V^ species. However, we were able to detect and quantify both As^III^ and As^V^ species in cultured human urothelial cells treated *in vitro* with iAs. We have shown that both MAs^III^ and DMAs^III^ are stable in these cells when stored at –80°C (Currier et al. 2011a, 2011b). To increase the feasibility of the analysis of MAs^III^ and DMAs^III^ in the small numbers of EUC that are typically available from a spot urine sample, we have recently replaced AAS in the HG-CT-AAS system with inductively coupled plasma–mass spectrometry (ICP-MS). The newly developed HG-CT-ICP-MS was reported to be suitable for the oxidation state-specific analysis of As in EUC, providing superior detection limits and high reproducibility (Matoušek et al. 2013).

The goal of the present study was to determine concentrations of As^III^ and As^V^ species in EUC from individuals exposed to iAs in drinking water and to examine the association between As species in EUC and the prevalence of diabetes.

## Materials and Methods

*The Chihuahua cohort*. All procedures involving human subjects were approved by institutional review boards at the University of North Carolina at Chapel Hill (UNC) and Cinvestav-IPN (Centro de Investigación y de Estudios Avanzados del Instituto Politécnico Nacional, Mexico City, Mexico). Individuals participating in the present study were among 1,163 men and women recruited for the Chihuahua cohort (Mexico); all study participants provided informed consent. This cohort was established between 2008 and 2013 to study chronic diseases associated with iAs exposure in drinking water. Only adults (≥ 18 years of age) with ≥ 5 years of uninterrupted residency in the study area were recruited. Pregnant women and participants who reported kidney or urinary tract infection were excluded because these conditions could affect the urinary pattern of iAs metabolites. Individuals with a potential for occupational exposure to As were also excluded. Data on residency, occupation, drinking-water sources and consumption, smoking, use of alcohol, drugs, or medication, and medical history were gathered at the time of enrollment using a questionnaire. Specific questions were asked about previous diagnosis of diabetes and the use of antidiabetic drugs. Samples of household tap water were collected for As analysis. The participants were then transported to Universidad Autónoma de Chihuahua to undergo a medical examination. Body weight and height were recorded and used to calculate BMI. A single spot urine sample was collected in sterile plastic cups and placed immediately on ice. Aliquots of urine samples were frozen and stored at –80°C for speciation analysis of As; the rest was used for EUC isolation. A single sample of fasting venous blood was drawn, followed by a standard oral glucose tolerance test (OGTT) in which a sample of venous blood was drawn 2 hr after an oral load of 75 g glucose. All blood samples were placed on ice immediately after collection. Plasma was isolated from the fasting and 2-hr blood by centrifugation at 4°C and frozen at –80°C.

*Isolation of EUC*. EUC were isolated from individuals recruited between March 2011 and August 2012. A total of 466 individuals underwent medical examination during this period; 428 provided urine for EUC isolation. EUCs were isolated from freshly collected urine (100 mL/participant) by centrifugation at 4°C. The cell pellet was washed with ice-cold phosphate-buffered saline (PBS) and again centrifuged. Cells were then resuspended in PBS, counted, and checked for the presence of bacteria, yeast, and red or white blood cells using a microscope. Only EUC free of microbial contamination and with < 5% of the total cell count represented by red or white blood cells (from a total of 374 participants) were used in the present study. All cells other than bacteria, yeast, and red or white blood cells were assumed, but not confirmed, to be EUC. EUC were stored at –80°C and shipped along with the urine samples on dry ice to UNC once per month for As speciation analysis.

*Diagnosis of diabetes*. Glucose levels in fasting and 2-hr plasma samples were measured using a Prestige 24i Chemistry Analyzer (Tokyo Boeki). The analyzer was calibrated before analysis, and reference human sera with normal and elevated glucose levels (Serodos and Serodos PLUS; Human Diagnostics Worldwide) were used for quality control. Study participants with fasting plasma glucose (FPG) ≥ 126 mg/dL or 2-hr plasma glucose (2HPG) ≥ 200 mg/dL, or with a self-reported doctor’s diagnosis or self-reported use of antidiabetic medication were classified as diabetic.

*Analyses of As in household water and urine*. Concentrations of As in acid-digested water samples were determined at Cinvestav-IPN using HG-CT-AAS (Del Razo et al. 2011). Urine samples were analyzed at UNC after storage at –80°C for up to approximately 1 month, which is known to result in oxidation of MAs^III^ and DMAs^III^ (Del Razo et al. 2011). Thus, only analysis of total iAs (iAs^III + V^), MAs (MAs^III + V^), and DMAs (DMAs^III + V^) was performed using HG-CT-AAS (Hernández-Zavala et al. 2008a). A certified standard reference material (SRM), Arsenic Species in Frozen Human Urine (SRM 2669; National Institute of Standards and Technology) was used with every shipment to assure accuracy. The concentrations of As species measured by HG-CT-AAS in SRM 2669 ranged from 86.7% to 106.4% of the certified values: 90.3–106.4% for iAs, 86.7–96.4% for MAs, and 88.2–99.0% for DMAs. The limits of detection (LODs) using 200 μL urine per sample were 0.05 ng As/mL for MAs or DMAs and 0.1 ng As/mL for iAs. The creatinine concentration in urine was determined by a colorimetric assay (Cayman Chemical Company); specific gravity was measured using a digital Atago PAL refractometer (Atago USA). It should be noted that the HG-CT-AAS cannot detect organic As species commonly found in seafood (e.g., arsenobetaine), and thus accounts for As species associated mainly with iAs exposure.

*Analyses of As species in EUCs*. As^III^ and As^V^ species in EUCs were analyzed at UNC using HG-CT-ICP-MS (Matoušek et al. 2013). Briefly, cell pellets were lysed in ice-cold deionized water. The trivalent species (As^III^, MAs^III^, and DMAs^III^) were measured in an aliquot of cell lysate directly, without pretreatment. Another aliquot was treated with 2% cysteine and analyzed for total iAs (iAs^III + V^), MAs (MAs^III + V^), and DMAs (DMAs^III + V^). The concentrations of iAs^V^, MAs^V^, and DMAs^V^ were determined as a difference between As^III + V^ values obtained for cysteine-treated aliquots and As^III^ values from untreated sample aliquots. For As^III^ species concentrations below LOD, the values of LOD divided by the square root of 2 were used when calculating the corresponding As^V^ values. Calibration curves were generated using cysteine-treated pentavalent As standards (at least 98% pure) as previously described (Hernández-Zavala et al. 2008a). The instrumental LODs for As species analyzed by HG-CT-ICP-MS ranged from 0.04 to 2.0 pg As. The analyses of As species in EUC were performed by a researcher who was unaware of the diabetes status of the individual study participant or of the As concentrations in the corresponding urine and water samples.

*Statistical analysis*. Continuous variables were described using means and SDs or medians and interquartile ranges (IQRs; for nonnormally distributed variables). Categorical variables were described using frequencies. For As species concentrations below LOD, the values of LOD divided by the square root of 2 were used for statistical analysis, including regression and descriptive analyses and to determine IQRs. The statistical significance of differences in characteristics of study participants with versus without diabetes was assessed using Student’s *t*-tests or one-way analyses of variance (ANOVA). Associations between As species in EUC and urine were estimated using linear regression models with log-transformed (log_10_) variables as well as with Spearman correlations. Associations of diabetes with concentrations of As species in EUC and urine were estimated using logistic regression to estimate odds ratios (ORs) and 95% confidence intervals (CIs). To control for potential confounding, sex (as a categorical variable) and age and BMI (as continuous variables) were included *a priori* as covariates. We also used linear regression models adjusted for age, sex, and BMI to estimate associations of log_10_-transformed FPG and 2HPG concentrations with concentrations of iAs metabolites and the sum of speciated As in urine. Age, sex, and BMI were included as covariates in these models. The linearity of the associations between As species and FPG/2HPG was assessed graphically and by linear regression using log_10_-transformed values. Slopes were significantly nonzero for all As species except pentavalent species in EUC. Unless otherwise specified, ORs, regression coefficients, and CIs are reported for a 1-IQR increment of exposure to facilitate comparison because of the different concentration ranges of As in EUCs and urine. Analyses of urinary metabolites of iAs were conducted both with and without urinary creatinine concentration or specific gravity adjustment. All statistical analyses were performed in Epi Info 7, version 1.0.6 (Centers for Disease Control and Prevention) and graphical representations were generated using GraphPad Instat software package (GraphPad Software Inc.). Statistical significance was considered at the level of *p* < 0.05.

## Results

*Basic characteristics of the study population*. The EUC samples free of microbial contamination and containing < 5% of red or white blood cells were obtained from a total of 374 participants (252 women and 122 men). More than 17% of these participants (17.5% of women and 18% of men) were classified as diabetic (Table 1) based on the FPG or 2HPG value or on their self-reported diabetes diagnosis or medication. Eleven participants who reported a previous diabetes diagnosis and/or taking antidiabetic medication (25.5% of those classified as diabetic) had FPG and 2HPG values in the normal or prediabetic range (i.e., FPG < 126 and 2HPG < 200 mg glucose/dL). Approximately 29% of the participants were overweight and 41% were obese. The average age and BMI were significantly higher among diabetic compared with nondiabetic individuals. No statistically significant differences between diabetic and nondiabetic individuals were found in the average EUC count, As concentration in drinking water, or sum of As species in urine (expressed either as nanograms of As per milliliter or nanograms of As per milligram creatinine). The average specific gravity of urine from diabetic individuals was significantly higher than in nondiabetic individuals, but the difference between the two groups in the sum of As species in urine adjusted for specific gravity was not statistically significant. The average sum of As species was higher in EUC from nondiabetic compared with diabetic participants; again, this difference was not statistically significant. Notably, basic characteristics of participants included in the present study and those of the entire Chihuahua cohort were very similar; however, the average age of the cohort was somewhat lower (see Supplemental Material, Table S1).

**Table 1:** Basic characteristics of the study participants [mean ± SD or *n* (%)].

Characteristic	Participants		
	All	Diabetic^*a*^	Non­diabetic
All participants	374 (100)	66 (17.6)	308 (82.4)
Females	252 (67.4)	44 (17.5)	208 (82.5)
Males	122 (32.6)	22 (18.0)	100 (82.0)
Age (years)	49.2 ± 15.6	56 ± 12.0*	48 ± 16.0*
As in drinking water (ppb)	55.2 ± 52.8	60.0 ± 50.9	54.1 ± 53.2
BMI	29.2 ± 6.1	30.8 ± 5.4*	28.9 ± 6.2*
26 ≤ BMI < 30	108 (29)	19 (29)	79 (26)
BMI ≥ 30	155 (41)	31 (47)	124 (40)
FPG (mg/dL)	95.9 ± 39.5	155.7 ± 62.8*	83.2 ± 12.1*
2HPG (mg/dL)	118.6 ± 60.4	204.9 ± 86.0*	100.4 ± 31.1*
FPG ≥ 126 mg/dL	38 (10.2)	38 (57.6)	0 (0)
2HPG ≥ 200 mg/dL	33 (8.8)	33 (50.0)	0 (0)
Self-reported diabetes diagnosis	43 (11.5)	43 (65.2)	0 (0)
Self-reported diabetes medication	30 (8.0)	30 (45.5)	0 (0)
EUC count in 100 mL urine^*b*^	374,008 ± 726,387	352,165 ± 468,388	378,689 ± 771,036
Sum of As species^*c*^ in EUC (pg As/10,000 cells)	127.7 ± 359.6	90.3 ± 202.1	135.7 ± 384.8
Sum of As species in urine (ng As/mL)	73.9 ± 73.2	82.0 ± 74.9	72.1 ± 72.9
Creatinine concentration in urine (mg/dL)	129.2 ± 90.2	127.1 ± 85.7	129.6 ± 91.3
Sum of As species in urine normalized for creatinine (ng As/mg creatinine)	65.7 ± 66.5	73.1 ± 77.9	64.2 ± 63.8
Specific gravity of urine	1.014 ± 0.007	1.017 ± 0.008*	1.014 ± 0.007*
Sum of As species in urine normalized for specific gravity (ng As/specific gravity unit)	109.7 ± 101.8	92.6 ± 62	113.4 ± 108.2
^***a***^Diabetic individuals were classified by either FPG ≥ 126 mg/dL, 2HPG ≥ 200 mg/dL, self-report of doctor diagnosis, or use of medication for treatment of diabetes. ^***b***^Neither red nor white blood cells were included in the cell counts. ^***c***^Sum of As species = iAs^V^ + iAs^III^ + MAs^V^ + MAs^III^ + DMAs^V^ + DMAs^III^. *p < 0.05, for continuous variables, comparing participants with and without diabetes by Student’s *t*-test.			

**Figure 1:**
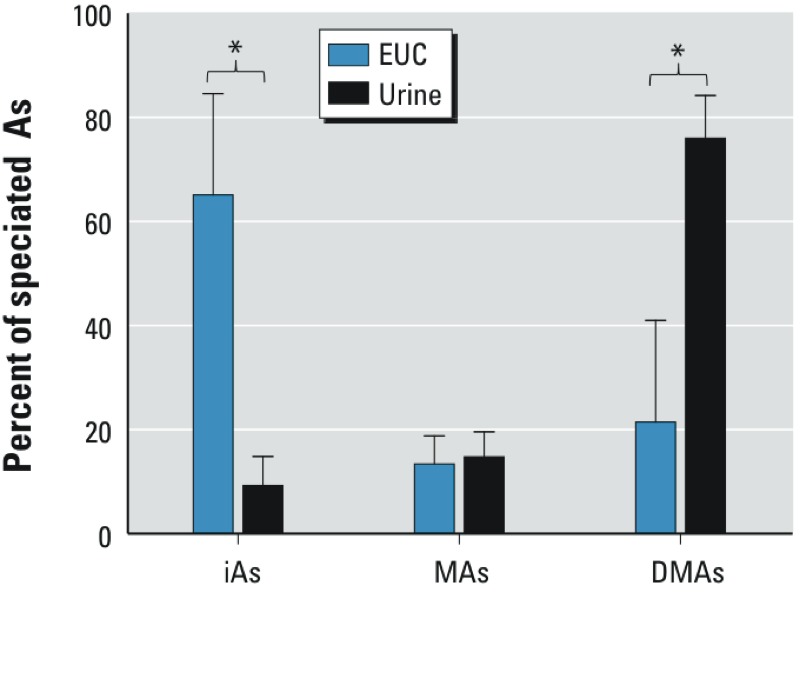
The composition of As species in EUC and urine. Data shown are mean + SD (*n* = 374). *p < 0.05, based on one-way ANOVA.

*Arsenic species in EUC and urine*. The speciation analysis of As was performed in all 374 samples of EUC and in the corresponding urine samples (Table 2). Concentrations of all As^III^ and As^V^ species were above LOD in 94% of EUC samples. In urine, concentrations of iAs and MAs were below LOD in 15 (4%) and 2 (0.5%) samples, respectively. DMAs was detected and quantified in all urine samples. There were marked differences in As speciation profiles in EUC and in urine (Figure 1). The ratios of DMAs/MAs and MAs/iAs were lower in EUC compared with urine. In urine, total DMAs (i.e., DMAs^III + V^) was the predominant species, accounting on average for 76% of all As species, with only 9% and 15% represented by iAs and MAs, respectively. In contrast, iAs^III^ and iAs^V^ were the major As species in EUC, representing on average 37% and 29% of speciated As. DMAs accounted for approximately 22% (2.4% for DMAs^III^ and 19.2% for DMAs^V^) and MAs for approximately 14% (MAs^III^ for 7.9% and MAs^V^ for 5.6%) of speciated As in EUC. In spite of the differences in As speciation profiles, statistically significant positive associations were found between the concentrations of individual As species in urine (not adjusted for creatinine) and EUC (see Supplemental Material, Figure S1). Notably, adjusting for creatinine had little or no effect on these associations (see Supplemental Material, Table S2).

**Table 2:** The concentrations of As species in EUC and urine.

As species	Minimum	25th percentile	Median	75th percentile	Maximum	IQR	Mean ± SD
EUC (pg As/10,000 cells)
iAs^III^	< LOD^*a*^	2.08	8.18	17.69	1,807	15.61	24.06 ± 103.0
MAs^III^	< LOD	0.45	1.78	4.04	151.7	3.59	4.17 ± 11.22
DMAs^III^	< LOD	0.16	0.41	1.54	141.3	1.38	2.73 ± 9.58
iAs^V^	< LOD	1.27	4.53	22.66	728.7	21.39	34.79 ± 86.26
MAs^V^	< LOD	0.19	0.85	5.08	776.2	4.89	13.11 ± 53.48
DMAs^V^	< LOD	0.66	1.90	13.82	2,303	13.16	49.18 ± 200.7
iAs^III + V^	0.36	6.35	17.13	41.11	2,148	34.76	58.52 ± 155.2
MAs^III + V^	0.04	1.12	3.14	9.84	803.3	8.72	17.25 ± 57.87
DMAs^III + V^	0.04	0.88	2.41	15.83	2,366	14.95	51.91 ± 208.9
Sum of As species^*b*^	0.78	10.05	25.50	76.3	3,773	66.25	127.7 ± 359.6
MAs/iAs	0.01	0.15	0.2	0.28	3.63	0.13	0.26 ± 0.28
DMAs/MAs	0.04	0.55	1.10	2.90	51.47	2.35	2.15 ± 3.64
DMAs/iAs	0.004	0.10	0.20	0.52	35.0	0.42	0.77 ± 2.5
(MAs + DMAs)/iAs	0.02	0.28	0.40	0.79	35.63	0.51	1.03 ± 2.68
Urine (ng As/mL)
iAs^III + V^	< LOD	0.98	4.62	10.21	119.2	9.23	7.3 ± 10.3
MAs^III + V^	< LOD	2.23	7.34	15.97	131.1	13.74	11.1 ± 12.9
DMAs^III + V^	0.36	13.05	40.46	82.72	307.2	69.67	55.42 ± 53.8
Sum of As species	0.52	17.00	53.50	108.4	492.5	91.40	73.87 ± 73.24
MAs/iAs	0.10	1.28	1.64	2.11	199.4	0.83	4.51 ± 18.1
DMAs/MAs	1.73	4.09	5.19	7.06	86.2	2.97	6.25 ± 5.27
DMAs/iAs	0.41	6.57	9.25	12.43	2,117	5.86	29.1 ± 142.3
(MAs + DMAs)/iAs	0.51	8.07	10.94	14.47	2,317	6.40	33.58 ± 159.6
^***a***^The minimum values were < LOD for iAs^III^ (*n* = 3), MAs^III^ (*n* = 13), DMAs^III^ (*n* = 19), iAs^V^ (*n* = 6), MAs^V^ (*n* = 21), or DMAs^V^ (*n* = 3) in EUC, and for iAs^III + V^ (*n* = 15) or MAs^III + V^ (*n* = 2) in urine. ^***b***^Sum of As species = iAs^V^ + iAs^III^ + MAs^V^ + MAs^III^ + DMAs^V^ + DMAs^III^.							

**Figure 2:**
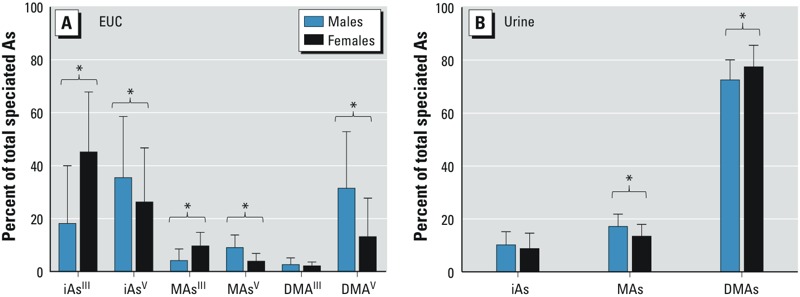
Differences in the composition of As species in EUC (*A*) and urine (*B*) collected from male and female study participants. Data shown are mean + SD (*n* = 252 for women; *n* = 122 for men). *p < 0.05 based on one-way ANOVA with Bonferroni’s posttest.

*EUC counts and As speciation in EUC and urine according to sex*. We found statistically significant differences in the numbers of EUC obtained from males and females. Cell counts in urine from males ranged from 450 to 2,128,000 cells/100 mL, compared with 1,800 to 9,717,000 cells/100 mL in samples from females. The mean EUC count (± SE) was 10 times higher in urine from women compared with men (529,258 ± 52,218/100 mL vs. 53,328 ± 18,782/100 mL). On average, EUC from females contained significantly less total speciated As ± SD than EUC from males: 40 ± 163 pg/10,000 cells versus 309 ± 542 pg/10,000 cells (see Supplemental Material, Figure S2B). For both males and females, statistically significant negative associations were found between the cell count and the concentration of total speciated As in EUC (see Supplemental Material, Figure S2). This association was stronger for males than females: β = –0.70 (95% CI: –0.82, –0.58; *r*^2^ = 0.54) versus β = –0.35 (95% CI: –0.46, –0.23; *r*^2^ = 0.12). Sex-related differences were also found in the composition of As species in both EUC and urine (Figure 2). iAs^III^ and MAs^III^ represented on average 43% and 10% of total speciated As in EUC from females, but only 18% and 4% in EUC from males (Figure 2A). On the other hand, the pentavalent As species—iAs^V^, MAs^V^, and DMAs^V^—accounted for greater proportions of As in EUC from males. Taken together, iAs species (iAs^III^ and iAs^V^) represented a smaller portion and DMAs species (DMAs^III^ and DMAs^V^) a greater portion of total speciated As in EUC from males compared with EUC from females. On average, males excreted significantly more As in urine as MAs^III + V^ (17%) than did females (14%), but the percentage of DMAs^III + V^ was smaller (73% vs. 78%) (Figure 2B). The DMAs/MAs ratio was smaller in male compared with female urines: 4.7 ± 1.84 versus 7.0 ± 6.16 (*p* < 0.0001).

**Figure 3:**
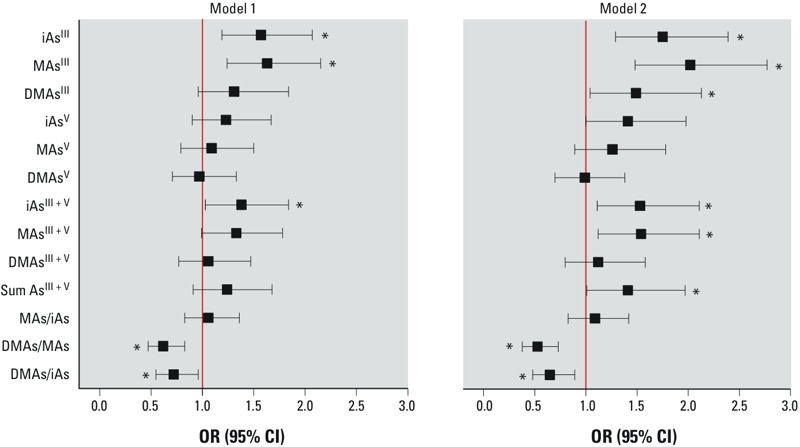
Association of diabetes with As species in EUC. In model 1, diabetes was classified by either FPG ≥ 126 mg/dL, 2HPG ≥ 200 mg/dL, or self-report of doctor diagnosis or use of medication for treatment of diabetes; in model 2, diabetes was classified only by FPG ≥ 126 mg/dL or 2HPG ≥ 200 mg/dL. ORs (95% CIs) are standardized to an increment of 1 IQR and adjusted for age, sex, and BMI. IQRs for each As species and species ratio are shown in Table 2. See Supplemental Material, Table S3, for numeric data. *p < 0.05.

*Associations of diabetes with As species in EUC and urine: logistic regression analysis.* The logistic regression analysis was performed using two models. Model 1 included all diabetic individuals as classified by FPG, 2HPG, or self-reported diagnosis or medication (*n* = 66). For model 2, only individuals with FPG ≥ 126 mg/dL or 2HPG ≥ 200 mg/dL were considered diabetic (*n* = 55); 11 individuals who reported diabetes diagnosis or medication but had both FPG < 126 mg/dL and 2HPG < 200 mg/dL were excluded. Both models were adjusted for age, sex, and BMI. Using either model, we found diabetes to be significantly associated with iAs^III^ and MAs^III^ concentrations in EUC—more strongly in model 2, with ORs for each IQR of 1.75 (95% CI: 1.29, 2.39) and 2.02 (95% CI: 1.48, 2.77), respectively (Figure 3; see also Supplemental Material, Table S3 for numeric data). In both models diabetes was negatively associated with the ratios of DMAs/MAs and DMAs/iAs in EUCs. Here again, the associations were stronger in model 2: OR = 0.53 (95% CI: 0.38, 0.73) and OR = 0.65 (95% CI: 0.48, 0.89), respectively. Diabetes was also positively associated with DMAs^III^ and with sum of As species in EUC, but these associations were statistically significant only for model 2 (OR = 1.49, 95% CI: 1.04, 2.13 and OR = 1.41, 95% CI: 1.01, 1.97, respectively). Diabetes was also positively associated with total iAs^III + V^ and MAs^III + V^. A marginally significant association was found with iAs^V^, but not with other As^V^ species. Notably, adjusting for cell count as a covariate in sensitivity analyses had no significant impact on the associations between As species in EUC and diabetes (data not shown). For example, ORs for model 2 were 1.81 (95% CI: 1.33, 2.48) for iAs^III^, 1.93 (95% CI: 1.42, 2.62) for MAs^III^, and 1.42 (95% CI: 1.02, 1.97) for DMAs^III^, thus practically matching the values obtained without the adjustment (see above).

**Figure 4:**
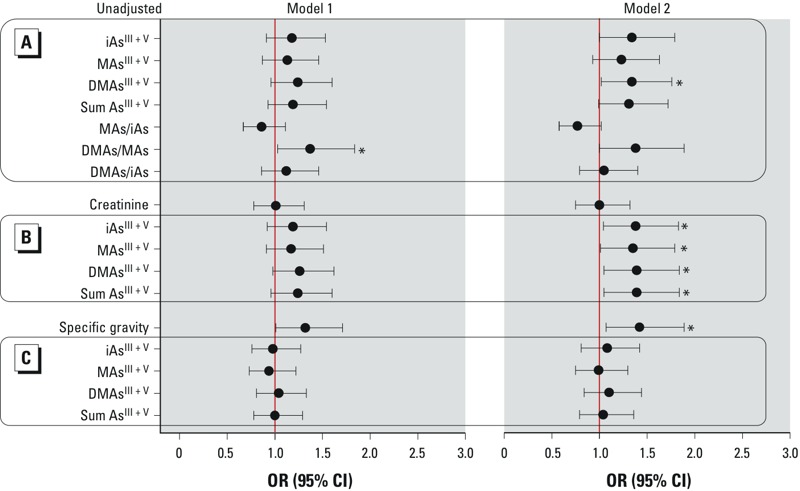
Association of diabetes with As species in urine, urine creatinine, and specific gravity. Arsenic species are either unadjusted (*A*) or adjusted for creatinine (*B*) or specific gravity (*C*). In model 1, diabetes was classified by either FPG ≥ 126 mg/dL, 2HPG ≥ 200 mg/dL, or self-report of doctor diagnosis or use of medication for treatment of diabetes; in model 2, diabetes was classified only by FPG ≥ 126 mg/dL or 2HPG ≥ 200 mg/dL. ORs (95% CIs) are standardized to an increment of 1 IQR and adjusted for age, sex, and BMI. IQRs for each As species and species ratio are indicated in Table 2. See Supplemental Material, Table S3, for numeric data. *p < 0.05.

In urine, total iAs, MAs, and DMAs and DMAs/MAs ratio were all positively associated with diabetes; however, these associations were statistically significant only for the DMAs/MAs ratio in model 1 (OR = 1.37, 95% CI: 1.03, 1.84) and total DMAs in model 2 (OR = 1.34, 95% CI: 1.02, 1.76) (Figure 4; see also Supplemental Material, Table S3 for numeric data). When adjusted for creatinine, total iAs, MAs, DMAs, and sum of As species in model 2 were all significantly associated with diabetes. In contrast, after adjusting for specific gravity all OR values were close to zero. Unlike creatinine, specific gravity itself was positively associated with diabetes: model 1, OR = 1.32 (95% CI: 1.01, 1.71); model 2, OR = 1.42 (95% CI: 1.07, 1.89).

*Associations of diabetes with As species in EUC and urine: linear regression analysis*. Linear regression analysis using logarithmically (log_10_) transformed FPG and 2HPG values was performed to further characterize associations between diabetes and As species in EUC and urine (Table 3). Both FPG and 2HPG were positively associated with the trivalent As species (iAs^III^, MAs^III^, DMAs^III^) and with the sum of As^III + V^ species in EUC (*p* < 0.01), but not significantly associated with the pentavalent As species in EUC. The ratios of DMAs/MAs and DMAs/iAs in EUCs were negatively associated with FPG and 2HPG (*p* ≤ 0.04). Statistically significant (*p* = 0.04) and marginally significant (*p* = 0.08) positive associations were found between the MAs/iAs ratio and FPG and 2HPG, respectively. FPG and 2HPG also were significantly associated with each of the As^III + V^ species in urine, regardless of adjustment for creatinine or specific gravity. Here again, specific gravity, but not creatinine, was positively associated with both FPG and 2HPG concentrations (*p* < 0.01). Both FPG and 2HPG were also positively associated with the urinary DMAs/MAs ratio.

**Table 3:** Associations of FPG and 2HPG with As species in EUCs and urine (adjusted for age, sex, and BMI).

As species	FPG			2HPG		
	β (95% CI)	*p*-Value	*r*^2^	β (95% CI)	*p*-Value	*r*^2^
EUC
iAs^III^	0.056 (0.038, 0.074)	< 0.01	0.16	0.042 (0.024, 0.060)	< 0.01	0.14
MAs^III^	0.062 (0.044, 0.080)	< 0.01	0.17	0.050 (0.032, 0.068)	< 0.01	0.15
DMAs^III^	0.050 (0.028, 0.072)	< 0.01	0.12	0.039 (0.017, 0.061)	< 0.01	0.12
iAs^V^	0.013 (–0.005, 0.031)	0.15	0.07	0.007 (–0.011, 0.025)	0.45	0.09
MAs^V^	0.015 (–0.001, 0.031)	0.08	0.08	0.010 (–0.006, 0.026)	0.21	0.09
DMAs^V^	0.010 (–0.008, 0.028)	0.25	0.07	0.007 (–0.011, 0.025)	0.40	0.09
iAs^III + V^	0.026 (0.012, 0.040)	< 0.01	0.11	0.022 (0.008, 0.036)	< 0.01	0.11
MAs^III + V^	0.035 (0.021, 0.049)	< 0.01	0.13	0.029 (0.015, 0.043)	< 0.01	0.13
DMAs^III + V^	0.015 (–0.001, 0.031)	0.06	0.08	0.014 (–0.002, 0.030)	0.08	0.09
Sum of As species	0.045 (0.023, 0.067)	< 0.01	0.11	0.035 (0.013, 0.057)	< 0.01	0.11
MAs/iAs	0.046 (0.003, 0.089)	0.04	0.08	0.039 (–0.004, 0.082)	0.08	0.09
DMAs/MAs	–0.072 (–0.101, –0.043)	< 0.01	0.12	–0.057 (–0.088, –0.026)	< 0.01	0.12
DMAs/iAs	–0.032 (–0.057, –0.007)	0.01	0.08	–0.033 (–0.064, –0.002)	0.04	0.10
(DMAs + MAs)/iAs	–0.014 (–0.045, 0.017)	0.40	0.07	–0.009 (–0.042, 0.024)	0.60	0.09
Urine (unadjusted)
iAs^III + V^	0.048 (0.030, 0.066)	< 0.01	0.13	0.043 (0.025, 0.061)	< 0.01	0.14
MAs^III + V^	0.062 (0.042, 0.082)	< 0.01	0.15	0.045 (0.023, 0.067)	< 0.01	0.13
DMAs^III + V^	0.078 (0.056, 0.100)	< 0.01	0.18	0.061 (0.039, 0.083)	< 0.01	0.15
Sum of As species	0.076 (0.054, 0.098)	< 0.01	0.17	0.059 (0.037, 0.081)	< 0.01	0.15
MAs/iAs	–0.001 (–0.038, 0.036)	0.95	0.02	–0.032 (–0.069, 0.005)	0.05	0.09
DMAs/MAs	0.072 (0.001, 0.143)	0.05	0.08	0.100 (0.029, 0.171)	< 0.01	0.11
DMAs/iAs	0.016 (–0.019, 0.051)	0.37	0.07	–0.005 (–0.040, 0.030)	0.80	0.09
(DMAs + MAs)/iAs	0.014 (–0.021, 0.049)	0.45	0.07	–0.009 (–0.044, 0.026)	0.62	0.09
Creatinine	0.033 (–0.006, 0.072)	0.09	0.07	0.005 (–0.034, 0.044)	0.79	0.09
Specific gravity	10.314 (6.257, 14.371)	< 0.01	0.13	5.995 (1.820, 10.170)	< 0.01	0.11
Urine (creatinine-adjusted)	
iAs^III + V^	0.052 (0.030, 0.074)	< 0.01	0.12	0.053 (0.031, 0.075)	< 0.01	0.15
MAs^III + V^	0.072 (0.048, 0.096)	< 0.01	0.15	0.060 (0.035, 0.085)	< 0.01	0.14
DMAs^III + V^	0.093 (0.032, 0.154)	< 0.01	0.18	0.083 (0.058, 0.108)	< 0.01	0.18
Sum of As species	0.091 (0.066, 0.116)	< 0.01	0.18	0.081 (0.056, 0.106)	< 0.01	0.17
Urine (specific gravity–adjusted)	
iAs^III + V^	0.040 (0.018, 0.062)	< 0.01	0.10	0.042 (0.020, 0.064)	< 0.01	0.13
MAs^III + V^	0.055 (0.031, 0.079)	< 0.01	0.12	0.042 (0.020, 0.064)	< 0.01	0.13
DMAs^III + V^	0.073 (0.048, 0.098)	< 0.01	0.14	0.065 (0.040, 0.090)	< 0.01	0.14
Sum of As species	0.070 (0.045, 0.095)	< 0.01	0.14	0.062 (0.037, 0.087)	< 0.01	0.14
Regression coefficients (βs) and 95% CIs are standardized to an increment of 1 IQR. *p*-Value for test of β = 0; linear regression model adjusted for age, sex, and BMI.						

## Discussion

The evidence linking chronic iAs exposure to diabetes was reviewed by a 2011 National Toxicology Program (NTP) workshop. This review concluded that existing data provide limited to sufficient support for an association of diabetes with high iAs exposures in drinking water (Maull et al. 2012). The workshop also discussed methods accounting for urine dilution when urinary As is used as an indicator of iAs exposure. Because of uncertainties associated with effects of iAs exposure or disease on urinary creatinine levels, the workshop review recommended that both raw and adjusted values should be reported (Maull et al. 2012). We followed this recommendation in the present study. We found all As^III + V^ species in urine to be positively associated with diabetes classified by FPG or 2HPG. When adjusting for creatinine, these associations were statistically significant in model 2; without the adjustment, significant association was found only with urinary DMAs^III + V^ (Figure 4; see also Supplemental Material, Table S3). Because of the uncertainty associated with urinary creatinine, adjusting for specific gravity has been recommended as an alternative method (Nermell et al. 2008). However, adjusting for specific gravity may bias associations with diabetes because urine of diabetic individuals, compared with healthy subjects, contains higher levels of albumin and glucose, resulting in higher specific gravity (Chadha et al. 2001; Voinescu et al. 2002). Indeed, we found a statistically significant positive association between diabetes and specific gravity and no associations between diabetes and urinary As species after adjusting for specific gravity.

The main goal of the present study was to examine associations between prevalent diabetes and As species in EUC as an alternative marker of iAs exposure and metabolism. Because cells provide a reducing environment, the toxic trivalent arsenicals are relatively stable even in samples stored for weeks (Currier et al. 2011a, 2011b). Our results show that As species profiles in EUC do not reflect those in urine. Specifically, iAs species are the major species in EUC but represent only a small percentage of As found in urine, whereas DMAs (i.e., DMAs^III + V^) is the major urinary metabolite. High iAs levels in EUC could be explained by formation of iAs-containing intracytoplasmic and intramitochondrial inclusions. These inclusions have recently been found in EUC from leukemia patients treated with arsenic trioxide (Wedel et al. 2013) and in bladder epithelium of mice exposed to iAs in drinking water (Dodmane et al. 2014). We also found that the concentrations and proportions of As species in EUC significantly differ between men and women. EUC from male donors contained approximately 10-fold more total speciated As than EUC from female donors; however, iAs^III^ and MAs^III^ accounted for smaller fractions of As in male compared with female EUC. These differences can probably be explained by different cell counts and cell types present in urine from males and females. The vesical trigone area of an adult female bladder is particularly susceptible to squamous metaplasia (Fortin et al. 2010; Tyler 1962), a noncancerous change in the epithelial lining that is ultimately responsible for greater numbers of cells in urine from females. Urine from females may also contain vaginal epithelial cells. However, the routine microscopy used in our study to examine EUC suspensions cannot reliably distinguish between epithelial cells of urothelial or squamous origin. Because of the relatively small numbers of cells collected from spot urines, we could not apply more sophisticated techniques (e.g., immunocytochemistry) to characterize the types and origin of EUC. Thus, for the purpose of this study we defined all cells isolated from the bacteria- and yeast-free urine containing < 5% of red or white blood cells as EUC.

We have previously reported that trivalent As species, particularly MAs^III^ and DMAs^III^, inhibit insulin-dependent glucose uptake by adipocytes (Paul et al. 2007) and glucose stimulated insulin secretion by isolated pancreatic islets (Douillet et al. 2013). Our recent study in the Zimapan and Lagunera regions of Mexico (Del Razo et al. 2011) indicated an association between the prevalence of diabetes and DMAs^III^ concentration in urine. In the present study, we found positive associations of diabetes with iAs^III^, MAs^III^, and DMAs^III^ concentrations in EUC, thus providing additional evidence that trivalent metabolites of iAs play important roles in the diabetogenic effects of iAs exposure. Notably, DMAs/MAs and DMAs/iAs ratios in EUC were inversely associated with diabetes. Although never before measured in EUC, the ratios of As species in urine have often been used in population studies to evaluate the body capacity to methylate iAs. Low DMAs/MAs and high MAs/iAs ratios in urine, as possible indicators of low methylation capacity, have been linked to increased risks of cancer and cardiovascular disease in populations chronically exposed to iAs exposure (Tseng 2007). In contrast, our study in Zimapan and Lagunera (Del Razo et al. 2011) and the present study show that in the case of diabetes, a high DMAs/MAs ratio in urine may be a risk factor.

## Conclusions

Our findings provide additional evidence for the association between diabetes and chronic exposure to iAs and for the role of the trivalent metabolites of iAs in the diabetogenic effects of this exposure. We also show that the speciation analysis of As in EUC—which facilitates assessment of trivalent species and avoids the need for dilution adjustments—can be used as an alternative to analysis of urinary As species. It should be noted, however, that in this study, the results obtained using the measures of As^III^ species in EUC were consistent with the results using measures of As^III + V^ species in urine after adjusting for creatinine. The associations between diabetes and As species in both EUC and urine were stronger when diabetes was classified only by FPG ≥ 126 or 2HPG ≥ 200 mg/dL, disregarding self-reported diagnosis or medication. This is consistent with a recent report from an American Indian cohort (Gribble et al. 2012) where an association between iAs exposure and diabetes was observed only among participants with poor diabetes control as indicated by elevated levels of glycated hemoglobin. Thus, using clinical indicators as compared with existing diabetes diagnosis may be required to better characterize the association of iAs exposure with prevalent or incident diabetes. The cross-sectional design, which cannot provide information on causality of the observed associations, and a modest sample size are the main limitations of the present study. Prospective studies in larger populations exposed to iAs, including the Chihuahua cohort, are needed to determine whether trivalent As species in EUC can be used to identify individuals or subpopulations who are at risk of developing diabetes as a result of chronic exposure to iAs.

## Supplemental Material

(1.1 MB) PDF

## References

[r1] Barr DB Wilder LC Caudill SP Gonzalez AJ Needham LL Pirkle JL 2005 Urinary creatinine concentrations in the U.S. population: implications for urinary biologic monitoring measurements. Environ Health Perspect 113 192 200; 10.1289/ehp.7337 15687057 PMC1277864

[r2] Boeniger MF, Lowry LK, Rosenberg J (1993). Interpretation of urine results used to assess chemical exposure with emphasis on creatinine adjustments: a review.. Am Ind Hyg Assoc J.

[r3] Chadha V, Garg U, Alon US (2001). Measurement of urinary concentration: a critical appraisal of methodologies.. Pediatr Nephrol.

[r4] Currier JM, Svoboda M, de Moraes DP, Matoušek T, Dědina J, Stýblo M (2011a). Direct analysis of methylated trivalent arsenicals in mouse liver by hydride generation-cryotrapping-atomic absorption spectrometry.. Chem Res Toxicol.

[r5] Currier JM, Svoboda M, Matoušek T, Dědina J, Stýblo M (2011b). Direct analysis and stability of methylated trivalent arsenic metabolites in cells and tissues.. Metallomics.

[r6] Del Razo M García-Vargas GG Valenzuela OL Hernandez-Castellanos E Sánchez-Peňa LC Drobná Z 2011 Exposure to arsenic in drinking water is associated with increased prevalence of diabetes: a cross-sectional study in the Zimapan and Lagunera regions in Mexico. Environ Health 10 73; 10.1186/1476-069X-10-73 21864395 PMC3169452

[r7] Dodmane PR, Arnold LL, Muirhead DE, Suzuki S, Yokohira M, Pennington KL (2014). Characterization of intracellular inclusions in the urothelium of mice exposed to inorganic arsenic.. Toxicol Sci.

[r8] Douillet C, Currier J, Saunders RJ, Bodnar WM, Matoušek T, Stýblo M (2013). Methylated trivalent arsenicals are potent inhibitors of glucose stimulated insulin secretion by murine pancreatic islets.. Toxicol Appl Pharmacol.

[r9] Fortin F, Anghel T, Brochu P, Lemieux N (2010). Optimizing urothelial cell preparation for the human urinary micronucleus assay.. Toxicol In Vitro.

[r10] Fu J Woods CG Yehuda-Shnaidman E Zhang Q Wong V Collins S 2010 Low-level arsenic impairs glucose-stimulated insulin secretion in pancreatic beta cells: involvement of cellular adaptive response to oxidative stress. Environ Health Perspect 118 864 870; 10.1289/ehp.0901608 20100676 PMC2898865

[r11] Gong ZL, Lu XF, Cullen WR, Le XC (2001). Unstable trivalent arsenic metabolites, monomethylarsonous acid and dimethylarsinous acid.. J Anal At Spectrom.

[r12] Gribble MO, Howard BV, Umans JG, Shara NM, Francesconi KA, Goessler W (2012). Arsenic exposure, diabetes prevalence, and diabetes control in the Strong Heart Study.. Am J Epidemiol.

[r13] Hernández-Zavala A, Matoušek T, Drobna Z, Paul DS, Walton F, Adair BM (2008a). Speciation analysis of arsenic in biological matrices by automated hydride generation-cryotrapping-atomic absorption spectrometry with multiple microflame quartz tube atomizer (multiatomizer).. J Anal At Spectrom.

[r14] Hernández-Zavala A Valenzuela OL Matoušek T Drobna Z Deˇdina J Garcia-Vargas GG 2008b Speciation of arsenic in exfoliated urinary bladder epithelial cells from individuals exposed to arsenic in drinking water. Environ Health Perspect 116 1656 1660; 10.1289/ehp.11503 19079716 PMC2599759

[r15] James KA, Marshall JA, Hokanson JE, Meliker JR, Zerbe GO, Byers TE (2013). A case-cohort study examining lifetime exposure to inorganic arsenic in drinking water and diabetes mellitus.. Environ Res.

[r16] Jerums G, Premaratne E, Panagiotopoulos S, Macisaac RJ (2010). The clinical significance of hyperfiltration in diabetes.. Diabetologia.

[r17] Kim NH, Mason CC, Nelson RG, Afton SE, Essader AS, Medlin JE (2013). Arsenic exposure and incidence of type 2 diabetes in southwestern American Indians.. Am J Epidemiol.

[r18] Kuo CC, Moon K, Thayer KA, Navas-Acien A (2013). Environmental chemicals and type 2 diabetes: an updated systematic review of the epidemiologic evidence.. Curr Diab Rep.

[r19] Mahalingaiah S Meeker JD Pearson KR Calafat AM Ye X Petrozza J 2008 Temporal variability and predictors of urinary bisphenol A concentrations in men and women. Environ Health Perspect 116 173 178; 10.1289/ehp.10605 18288314 PMC2235217

[r20] Matoušek T, Currier JM, Trojánková N, Saunders RJ, Ishida MC, González-Horta C (2013). Selective hydride generation- cryotrapping- ICP-MS for arsenic speciation analysis at picogram levels: analysis of river and sea water reference materials and human bladder epithelial cells.. J Anal At Spectrom.

[r21] Maull EA Ahsan H Edwards J Longnecker MP Navas-Acien A Pi J 2012 Evaluation of the association between arsenic and diabetes: a National Toxicology Program Workshop Review. Environ Health Perspect 120 1658 1670; 10.1289/ehp.1104579 22889723 PMC3548281

[r22] Nermell B, Lindberg AL, Rahman M, Berglund M, Persson LA, El Arifeen S (2008). Urinary arsenic concentration adjustment factors and malnutrition.. Environ Res.

[r23] Pan WC, Seow WJ, Kile ML, Hoffman EB, Quamruzzaman Q, Rahman M (2013). Association of low to moderate levels of arsenic exposure with risk of type 2 diabetes in Bangladesh.. Am J Epidemiol.

[r24] Paul DS, Devesa V, Hernández-Zavala A, Adair BM, Walton FS, Drobna B, et al. (2008). Environmental arsenic as a disruptor of insulin signaling.

[r25] Paul DS Harmon AW Devesa V Thomas DJ Stýblo M 2007 Molecular mechanisms of the diabetogenic effects of arsenic: inhibition of insulin signaling by arsenite and methylarsonous acid. Environ Health Perspect 115 734 742; 10.1289/ehp.9867 17520061 PMC1867998

[r26] Tseng CH (2007). Arsenic methylation, urinary arsenic metabolites and human diseases: current perspective.. J Environ Sci Health C Environ Carcinog Ecotoxicol Rev.

[r27] Tyler DE (1962). Stratified squamous epithelium in vesical trigone and urethra—findings correlated with menstrual cycle and age.. Am J Anat.

[r28] Voinescu GC, Shoemaker M, Moore H, Khanna R, Nolph KD (2002). The relationship between urine osmolality and specific gravity.. Am J Med Sci.

[r29] Wang W, Xie Z, Lin Y, Zhang D (2014). Association of inorganic arsenic exposure with type 2 diabetes mellitus: a meta-analysis.. J Epidemiol Community Health.

[r30] Wedel WR, Muirhead DE, Arnold LL, Dodmane PR, Lele SM, Maness-Harris L (2013). Urothelial cell intracytoplasmic inclusions after treatment of promyelocytic leukemia with arsenic trioxide.. Toxicol Sci.

