# Supplementary material for: Associations between Arsenic Species in Exfoliated Urothelial Cells and Prevalence of Diabetes among Residents of Chihuahua, Mexico
Source: Environ Health Perspect. 2014 Jun 27;122(10):1088–94. doi: 10.1289/ehp.1307756 (PMC4181927; doi:10.1289/ehp.1307756)
Supplement: (1.1 MB) PDF [file ehp.1307756.s001.508.pdf]

Environmental Health Perspectives doi:10.1289/ehp.1307756

Supplemental Material

Associations between Arsenic Species in Exfoliated Urothelial Cells and Prevalence of Diabetes among Residents of Chihuahua, Mexico

Jenna M. Currier, María C. Ishida, Carmen González-Horta, Blanca Sánchez-Ramírez, Lourdes Ballinas-Casarrubias, Daniela S. Gutiérrez-Torres, Roberto Hernández Cerón, Damián Viniegra Morales, Francisco A. Baeza Terrazas, Luz M. Del Razo, Gonzalo G. García-Vargas, R. Jesse Saunders, Zuzana Drobná, Rebecca C. Fry, Tomáš Matoušek, John B. Buse, Michelle A. Mendez, Dana Loomis, and Miroslav Stýblo

Table S1. Basic characteristics of participants included in the present study and in the entire Chihuahua cohort 2

Figure S1. Associations between the concentrations of As species in exfoliated urothelial cells (EUC) and urine 3

Table S2. Associations between the concentrations of As species in EUC and As species in urine normalized for creatinine 4

Figure S2. Associations between the EUC count and As content 5

Table S3. Associations of diabetes with As species in EUC and urine 6

Table S1. Basic characteristics of participants included in the present study and in the entire Chihuahua cohort

Characteristics	Present study	Chihuahua cohort
All subjects (N)	374	1163
Females (%)	67.4	67.0
Age, years<br>(range, mean ± SD)	18–90<br>49.2 ± 15.6*	18–90<br>45.7 ± 15.8
As in water, ppb<br>(range, median)	0.01–275<br>48.7	< LOD–420[a]<br>47.4
Sum of As species in urine, ppb<br>(range, median)	0.5–492<br>53.5	0.5–375[b]<br>53.2
BMI > 30 (%)	41	40
Diabetic subjects (%)[c]	17.6	17.3

[a]To date, As concentrations were determined only in 876 samples of drinking water. [b]To date, concentrations of As species were determined only in 939 samples of urine. [c]Diabetes is classified by FPG ≥ 126 mg/dL or 2HPG ≥ 200 mg/dL, or self-reported doctor's diagnosis or use of anti-diabetic medication (based on the questionnaire data).

*Difference between the present study and the Chihuahua cohort is statistically significant ($p < 0.05$).

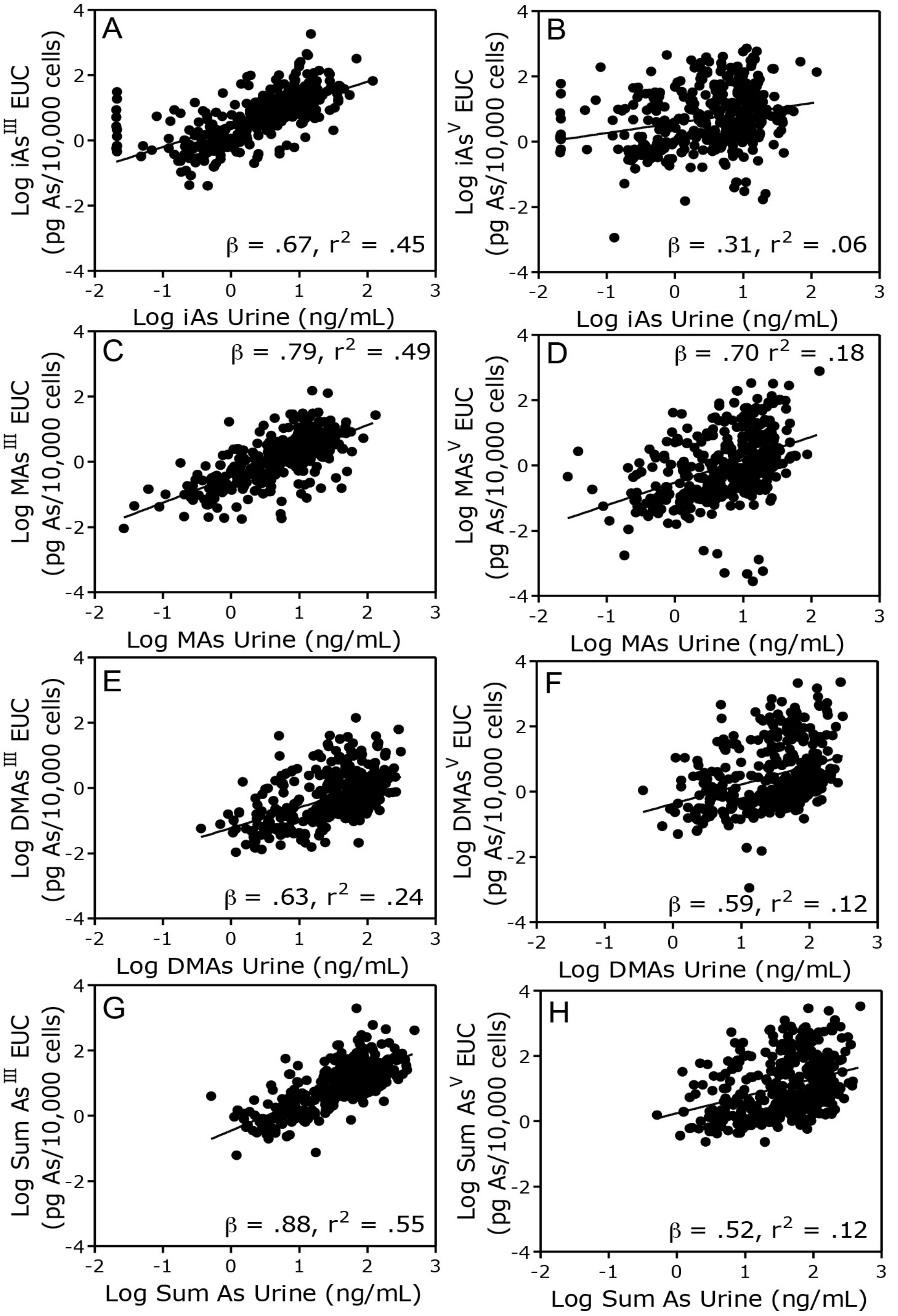

Figure S1. Associations between the log_{10}-transformed concentrations of As species in EUC and log_{10}-transformed As species in urine (not adjusted for creatinine): A, iAs^{III} in EUC vs. iAs^{III+V} in urine; B, iAs^{V} in EUC vs. iAs^{III+V} in urine; C, MAs^{III} in EUC vs. MAs^{III+V} in urine; D, MAs^{V} in EUC vs. MAs^{III+V} in urine; E, DMAs^{III} in EUC vs. DMAs^{III+V} in urine; DMAs^{V} in EUC vs. DMAs^{III+V} in urine; G, sum of As^{III} species in EUC vs. sum of As^{III+V} species in urine; H sum of As^{V} species in EUC vs. sum of As^{III+V} species in urine; Slope (β) and correlation coefficient (r^2) determined by linear regression analysis are shown. All slopes are significantly different from 0 ($p < 0.001$).

Table S2. Associations between the log_{10}-transformed concentrations of As species in EUC and log_{10}-transformed As species in urine after adjustment for urinary creatinine.

As species in urine	As species in EUC	β ± SE	r^2
iAs^{III+V}	iAs^{III}	0.70 ± 0.048	0.36
iAs^{III+V}	iAs^{V}	0.33 ± 0.073	0.05
MAs^{III+V}	MAs^{III}	0.87 ± 0.054	0.41
MAs^{III+V}	MAs^{V}	0.79 ± 0.094	0.16
$DMAs^{III+V}$	$DMAs^{III}$	0.64 ± 0.073	0.17
$DMAs^{III+V}$	$DMAs^{V}$	0.52 ± 0.103	0.06
Sum of As^{III+V} species	Sum of As^{III} species	0.96 ± 0.056	0.44
Sum of As^{III+V} species	Sum of As^{III} species	0.53 ± 0.090	0.09

Slope (β), standard error (SE) and correlation coefficient (r^2) determined by linear regression analysis are shown. All slopes are significantly different from 0 ($p < 0.001$).

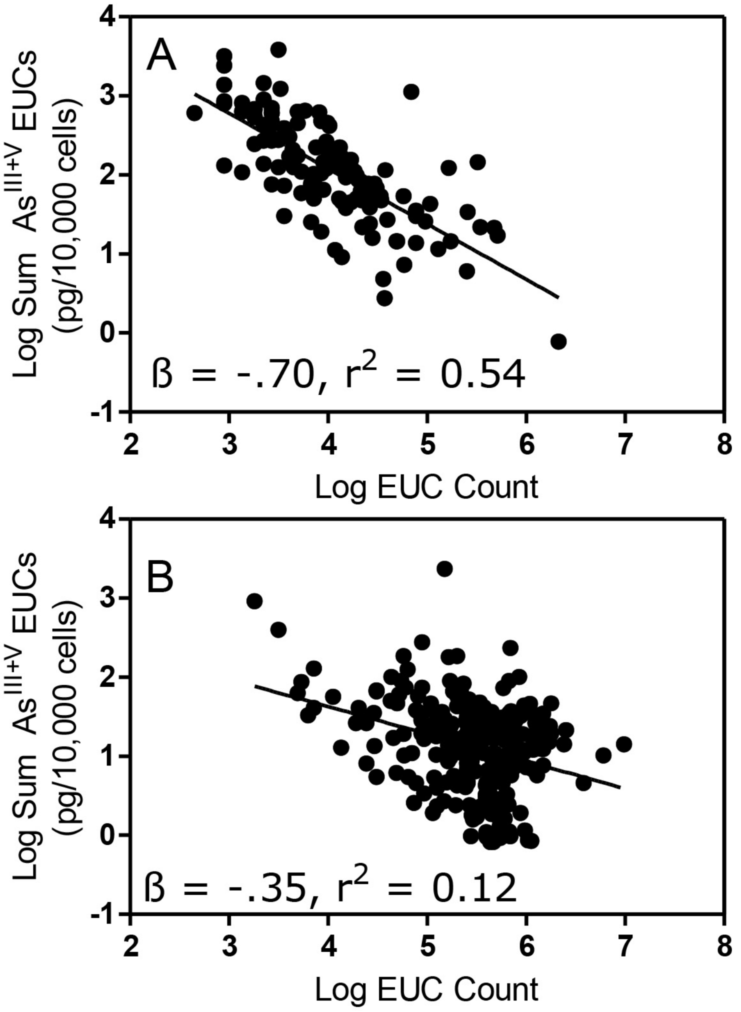

Figure S2. Associations between the log-transformed EUC count and As content (log-transformed sum of As species) for EUC samples obtained from male (A) and female (B) study participants. Slope (β) and correlation coefficient (r^2) determined by linear regression analysis are shown. Both slopes are significantly different from 0 ($p < 0.05$).

Table S3. Associations of diabetes with As species in EUC and urine.

As species	MODEL 1[a] OR (95% CI)[c]	MODEL 1 *p*	MODEL 2[b] OR (95%CI)	MODEL 2 *p*
**EUC**				
iAs^{III}	1.57 (1.19, 2.07)	< 0.01	1.75 (1.29, 2.39)	< 0.01
MAs^{III}	1.63 (1.24, 2.15)	< 0.01	2.02 (1.48, 2.77)	< 0.01
$DMAs^{III}$	1.31 (0.96, 1.84)	0.09	1.49 (1.04, 2.13)	0.03
iAs^{V}	1.23 (0.90, 1.67)	0.20	1.41 (1.00, 1.98)	0.05
MAs^{V}	1.09 (0.79, 1.50)	0.61	1.26 (0.89, 1.78)	0.20
$DMAs^{V}$	0.97 (0.71, 1.33)	0.85	0.99 (0.70, 1.38)	0.94
iAs^{III+V}	1.38 (1.03, 1.84)	0.03	1.53 (1.11, 2.11)	< 0.01
MAs^{III+V}	1.33 (0.99, 1.78)	0.06	1.54 (1.12, 2.11)	< 0.01
$DMAs^{III+V}$	1.06 (0.77, 1.47)	0.70	1.12 (0.80, 1.58)	0.50
Sum of As species[d]	1.24 (0.91, 1.68)	0.17	1.41 (1.01, 1.97)	0.04
MAs/iAs	1.06 (0.83, 1.36)	0.63	1.09 (0.83, 1.42)	0.54
DMAs/MAs	0.62 (0.47, 0.83)	< 0.01	0.53 (0.38, 0.73)	< 0.01
DMAs/iAs	0.72 (0.55, 0.96)	0.02	0.65 (0.48, 0.89)	0.01
(DMAs+MAs)/iAs	0.77 (0.56, 1.04)	0.08	0.78 (0.56, 1.05)	0.09
**Urine (unadjusted)**				
iAs^{III+V}	1.18 (0.91, 1.53)	0.22	1.34 (1.00, 1.79)	0.05
MAs^{III+V}	1.13 (0.87, 1.46)	0.36	1.23 (0.93, 1.63)	0.14
$DMAs^{III+V}$	1.24 (0.96, 1.60)	0.10	1.34 (1.02, 1.76)	0.04
Sum of As species	1.19 (0.93, 1.54)	0.17	1.31 (0.99, 1.72)	0.06
MAs/iAs	0.86 (0.67, 1.11)	0.25	0.77 (0.58, 1.02)	0.07
DMAs/MAs	1.37 (1.03, 1.84)	0.03	1.38 (1.00, 1.89)	0.05
DMAs/iAs	1.12 (0.86, 1.46)	0.38	1.05 (0.79, 1.40)	0.74
(DMAs+MAs)/iAs	0.99 (0.75, 1.30)	0.95	1.02 (0.77, 1.34)	0.91
Creatinine	1.01 (0.78, 1.31)	0.93	1.00 (0.75, 1.32)	0.98
Specific gravity	1.32 (1.01, 1.71)	0.07	1.42 (1.07, 1.89)	0.02
**Urine (creatinine adjusted)**				
iAs^{III+V}	1.19 (0.92, 1.54)	0.19	1.38 (1.04, 1.83)	0.03
MAs^{III+V}	1.17 (0.91, 1.51)	0.23	1.35 (1.01, 1.79)	0.04
$DMAs^{III+V}$	1.26 (0.98, 1.62)	0.08	1.39 (1.05, 1.84)	0.02
Sum of As species	1.24 (0.96, 1.60)	0.10	1.39 (1.05, 1.84)	0.02
**Urine (specific gravity adjusted)**				
iAs^{III+V}	0.98 (0.76, 1.27)	0.87	1.08 (0.81, 1.42)	0.61
MAs^{III+V}	0.94 (0.73, 1.22)	0.65	0.99 (0.75, 1.30)	0.92
$DMAs^{III+V}$	1.04 (0.81, 1.33)	0.78	1.10 (0.84, 1.44)	0.51
Sum of As species	1.00 (0.78, 1.29)	0.99	1.04 (0.79, 1.36)	0.79

[a]Model 1: Diabetes classified by either FPG $\geq$ 126 mg/dL, 2HPG $\geq$ 200 mg/dL, self-reported doctor's diagnosis or use of medication to treat diabetes. [b]Model 2: Diabetes classified only by FPG $\geq$ 126 mg/dL or 2HPG $\geq$ 200 mg/dL. [c]Odds ratio (OR) and 95% confidence interval (CI) are standardized to an increment of one inter-quartile range (IQR) and adjusted for age, sex, and BMI (IQRs are listed in Table 2). [d]Sum of As species = iAs^{V} + iAs^{III} + MAs^{V} + MAs^{III} + $DMAs^{V}$ + $DMAs^{III}$.
